# The Straw That Broke the Camel’s Back: Natural Variations in 17β-Estradiol and COMT-Val158Met Genotype Interact in the Modulation of Model-Free and Model-Based Control

**DOI:** 10.3389/fnbeh.2021.658769

**Published:** 2021-07-08

**Authors:** Esther K. Diekhof, Andra Geana, Frederike Ohm, Bradley B. Doll, Michael J. Frank

**Affiliations:** ^1^Neuroendocrinology and Human Biology Unit, Department of Biology, Faculty of Mathematics, Informatics and Natural Sciences, Institute of Zoology, Universität Hamburg, Hamburg, Germany; ^2^Department of Cognitive, Linguistic, and Psychological Sciences, Brown University, Providence, RI, United States; ^3^Carney Institute for Brain Science, Brown University, Providence, RI, United States; ^4^New York University, New York, NY, United States; ^5^Columbia University, New York, NY, United States

**Keywords:** reinforcement learning, estrogen, menstrual cycle, dopamine, reward learning, reward volatility, COMT-Valcpsdummy158Met genotype

## Abstract

The sex hormone estradiol has recently gained attention in human decision-making research. Animal studies have already shown that estradiol promotes dopaminergic transmission and thus supports reward-seeking behavior and aspects of addiction. In humans, natural variations of estradiol across the menstrual cycle modulate the ability to learn from direct performance feedback (“model-free” learning). However, it remains unclear whether estradiol also influences more complex “model-based” contributions to reinforcement learning. Here, 41 women were tested twice – in the low and high estradiol state of the follicular phase of their menstrual cycle – with a Two-Step decision task designed to separate model-free from model-based learning. The results showed that in the high estradiol state women relied more heavily on model-free learning, and accomplished reduced performance gains, particularly during the more volatile periods of the task that demanded increased learning effort. In contrast, model-based control remained unaltered by the influence of hormonal state across the group. Yet, when accounting for individual differences in the genetic proxy of the COMT-Val158Met polymorphism (rs4680), we observed that only the participants homozygote for the methionine allele (*n* = 12; with putatively higher prefrontal dopamine) experienced a decline in model-based control when facing volatile reward probabilities. This group also showed the increase in suboptimal model-free control, while the carriers of the valine allele remained unaffected by the rise in endogenous estradiol. Taken together, these preliminary findings suggest that endogenous estradiol may affect the balance between model-based and model-free control, and particularly so in women with a high prefrontal baseline dopamine capacity and in situations of increased environmental volatility.

## Introduction

Neuroactive steroid hormones like 17β-estradiol (estradiol) are important modulators of neural processing (Becker, 2016). As a natural dopamine agonist, estradiol has been implicated in reward processing and basic aspects of reinforcement learning and may modulate activity in the associated frontostriatal circuits (Sakaki and Mather, 2013; Diekhof, 2018). In the striatum, estradiol modulates dopaminergic transmission, which increases the incentive salience of immediate reward and promotes the development of behavioral habits that are inflexible and difficult to overcome. This is why estradiol may also play a central role in the initiation and reinstatement of female addiction (Becker, 2016). However, estradiol is also involved in higher-order prefrontal functions such as working memory (Dumas et al., 2010; Jacobs and D’Esposito, 2011; Hampson and Morley, 2013). This suggests that estradiol could contribute to the more goal-directed aspects of the decision-making process, which enable more structured choices that can override existing habits (Daw et al., 2005). However, this association hasn’t been assessed yet and it remains elusive to what extent variations in estradiol influence higher-order cognitive operations during the decision-making process.

Decision-making in complex environments involves different learning strategies. Action selection can be based on previous performance feedback. However, such a “model-free” strategy that requires agents to simply repeat actions that are reinforcing is too inflexible to account for more complex cognitive strategies. These latter strategies become necessary in more structured decision environments that for instance require the prospective anticipation of action consequences using a previously learned map or model (Doll et al., 2015). Recent accounts on reinforcement learning theory thus propose a dual-architecture of two functionally distinct computational processes in decision-making, thereby dissociating “model-free” and “model-based” control (Daw et al., 2005; Dolan and Dayan, 2013; Daw and Dayan, 2014). This dual-architecture is based on the assumption that optimization of reward outcome does not always depend on the most recent choice, but may require taking into consideration the most likely cause of a reward and to do so the learner must represent the task structure. Therefore, a second functionally distinct computational process, “model-based” control, has been proposed to support sequential choice that combines short-term predictions of immediate actions in a sequence of choices that are used to build a prospective model of the world. The process of model-based control is assumed to capture the overall complexity of the environment beyond model-free learning and enables reflective planning (Daw et al., 2005). In humans, the relative dominance of model-based control over the model-free system is correlated with other higher-order cognitive operations like declarative memory (Doll et al., 2015), working-memory span (Potter et al., 2017), and attentional control (Otto, 2013).

Model-free control is believed to rely on the prediction error signal of mesencephalic dopamine neurons (Glimcher, 2011). Transient changes in dopamine thereby signal the difference between received and predicted reward in the striatum, where these signals have opposing effects on the dopamine D1- and D2-receptors, i.e., DRD1 and DRD2, respectively, as well as on the associated processes of approach and avoidance learning (Collins and Frank, 2014). Model-based control depends on central dopamine that modulates activation in both the striatum and the prefrontal cortex. Deserno et al. (2015) found that a higher presynaptic dopamine level in the ventral striatum was associated with a bias toward model-based learning and promoted model-based activation in the lateral prefrontal cortex at the expense of model-free prediction errors in the ventral striatum. Further, the transient enhancement of central dopamine by agonist treatment enhanced model-based learning capacity in healthy young men (Wunderlich et al., 2012; but see Kroemer et al., 2019 for a null finding). In contrast, reductions in the ability to rely on the model-based component have been found in male addicts with a disturbed dopamine system (Sebold et al., 2014), in Parkinson patients in the dopamine-deprived state (Sharp et al., 2016), and following disruptions of the prefrontal cortex by transcranial magnetic stimulation (Smittenaar et al., 2013). These observations fit with the idea that prefrontal and striatal dopaminergic mechanisms interact in higher-order cognitive operations, such as model-based learning, both supporting the stabilization and flexible updating of goal representations (Frank and O’Reilly, 2006; Cools and D’Esposito, 2011).

The COMT-Val158Met polymorphism codes for the activity of the dopamine-degrading enzyme catechol-o-methyltransferase (COMT) (Apud et al., 2007; Käenmäki et al., 2010), which is more active in the prefrontal cortex of carriers of the Val allele than of individuals homozygote for the Met allele. This may lead to higher prefrontal dopamine availability in Met-homozygotes (see also Schacht, 2016). It has been proposed that the Met allele is more beneficial for the stabilization of prefrontal information processing and may protect goal-directed information from interference, supposedly by optimizing signaling through prefrontal DRD1 in relation to DRD2. In contrast, homozygosity for the Val allele may predict less balanced signaling through these receptors, which results in reduced cognitive capacity (Durstewitz and Seamans, 2008; Slifstein et al., 2008; Schacht, 2016). The COMT-Val158Met polymorphism has been associated with model-based control (Doll et al., 2016), as well as with other aspects of higher-order cognition including working memory and executive function (Mier et al., 2010; Schacht, 2016). Homozygosity for the Met allele thereby predicted an overall advantage in prefrontal tasks, especially those with increased cognitive load (Mier et al., 2010).

As an indicator of dopamine baseline capacity in the prefrontal cortex, the COMT-Val158Met polymorphism has further been observed to interact with (pharmacological) agents that transiently enhance dopamine. Notably, the resulting relationship between the combined effect of tonic and phasic dopamine on cognitive performance was not linear, but rather followed an inverted U-shape. This has led to the “Inverted-U-Hypothesis,” which presumes that peak cognitive performance is linked to an optimal dopamine level that lies in the intermediate physiological range, while cognitive performance is believed to decline in individuals with either higher or lower than this optimal dopamine range, which has been shown repeatedly (Cools and D’Esposito, 2011). Therefore, in the present study we decided to account for the COMT-Val158Met polymorphism as a baseline marker of prefrontal dopamine, when assessing the phasic influence of estradiol on model-based control.

Apart from baseline differences in dopamine, our study assessed the role of estradiol as a natural dopamine agonist in model-based reinforcement learning. In rodents, estradiol modulates dopamine within frontostriatal networks. Estradiol increases dopaminergic transmission and amplifies the reward-related dopamine release, for example by augmenting DRD1 action, while concurrently suppressing DRD2 action (Lévesque et al., 1989; Krentzel and Meitzen, 2018; see also Becker, 2016; Yoest et al., 2018 for review). Similarly, estradiol down-regulates the dopamine-degrading enzyme COMT in the female prefrontal cortex, which in turn increases dopamine content in this structure (Xie et al., 1999; Schendzielorz et al., 2011). For these reasons, we expected an interaction between baseline dopamine and the phasic influence of estradiol in women, when comparing distinct high and low estradiol phases of the natural menstrual cycle. We decided to test women twice during the follicular phase. During the follicular phase estradiol level rises from its nadir until it reaches its cyclic peak right before ovulation. Progesterone, another steroid hormone important for female reproductive function, remains at a low concentration throughout the follicular phase. In the second half of the menstrual cycle, estradiol rises again toward the mid luteal phase. But this time, progesterone concentration is also increased (Sakaki and Mather, 2013). This is insofar important, since progesterone inhibits dopaminergic transmission through various physiological mechanisms, and could thus antagonize the dopamine agonistic effect of estradiol during the luteal phase (e.g., Luine and Rhodes, 1983; Dluzen and Ramirez, 1984, 1987; Luine and Hearns, 1990). By restricting our tests to the early (low estradiol) and late (high estradiol) follicular phase, we were able to assess the effect of the dopamine agonist estradiol widely uncontaminated by the dopamine antagonist progesterone.

In line with the dopamine-agonistic properties of estradiol, previous studies with humans showed that estradiol influenced model-free learning, also in interaction with the dopaminergic baseline capacity of the striatum that followed an inverted U-shape relationship (Diekhof, 2015; Jakob et al., 2018; see also Diekhof, 2018). In one study reward sensitivity was compromised when estradiol level reached its peak in the late follicular phase of the menstrual cycle. Conversely, intermediate estradiol levels at the beginning of the follicular phase promoted reward sensitivity. This was especially true for individuals with a lower dopamine baseline capacity in the striatum (Diekhof, 2015), as indicated by lower trait impulsivity (see also Buckholtz et al., 2010). In a similar vein, Jakob et al. (2018) observed that carriers of the 9-repeat-allele of the DAT1 genotype, with a higher dopamine transporter (DAT) density in the striatum, apparently experienced a marked reversal of DAT function as a consequence of rising estradiol, which led to a significant decline in the capacity to avoid negative outcomes in the high estradiol phase.

In the human prefrontal cortex, estradiol has been found to stabilize working memory representations, most likely also through its interaction with dopamine, and particularly so in situations of high cognitive demand (Dumas et al., 2010; Hampson and Morley, 2013). One prominent finding also supported the “Inverted-U-Hypothesis,” by demonstrating that the effect of estradiol on working memory performance and prefrontal activity depended on baseline dopamine concentration, and particularly so in high-load conditions (Jacobs and D’Esposito, 2011). A dose-dependency of estradiol could further be observed in ovariectomized rats in that only a moderate dose, but neither a low nor high dosage of estradiol preserved cognitive performance under high-load working memory demands (Bimonte and Denenberg, 1999). This shows that even independent of tonic dopamine, a deficit or abundance of estradiol could destabilize prefrontal working memory representations.

Until now, neurocognitive research has only addressed the role of estradiol in model-free learning (Diekhof, 2018). Considering the modulatory influence of estradiol on frontostriatal networks and dopamine (Becker, 1999; Yoest et al., 2018), and its association with both probabilistic feedback learning (e.g., Diekhof, 2015) and higher-order working memory processes (e.g., Jacobs and D’Esposito, 2011), we hypothesized that estradiol – as a natural dopamine-agonist – should also modulate model-based learning. The major aim of the present study was to examine whether model-based reinforcement learning is affected by the high estradiol state of the late follicular phase compared to the low estradiol state at the beginning of the follicular phase. Further, we also assessed whether the hypothesized effect of estradiol on model-based learning depends on prefrontal dopaminergic baseline capacity, similar to what has been demonstrated for model-free control in relation to striatal dopamine (Diekhof, 2018).

For this purpose, 41 women performed a Two-Step Markov Decision Task (TS-task), once in the low estradiol state of the early follicular phase and once during the high estradiol state of the late follicular phase. The TS-task combined features of a sequential choice task and a probabilistic selection task, which allowed us to assess model-based relative to model-free choice, while participants tried to maximize overall gain (Doll et al., 2016). Each of the 300 experimental trials consisted of two consecutive decision stages. At the initial stage of the TS-task, the participants had to decide between two arbitrary stimuli (a pair of Sanskrit symbols). The initial decision for one of the symbols then stochastically determined a set of second-stage options, i.e., one of the two second-stage stimulus pairs, with fixed transition probabilities (0.7 and 0.3). Depending on the initial choice, one set of options at the second-stage occurred more often, i.e., the “common transition” occurred in 70% of selections of the given first-stage symbol. The other second-stage set is denoted as the “rare transition” that occurred only in 30% of a given first-stage selection. After the selection of a symbol at the second stage, subjects received feedback, either in form of a monetary token or a feedback indicating outcome omission. The outcome was probabilistic. In the first 150 trials (the “drift phase”), outcome probability was slowly and randomly drifting between 0.25 and 0.75, while in the remaining 150 trials (the “stable phase”) the reward probabilities for each of the two second-stage sets reached their final values, which was 0.7:0.3 for one and 0.6:0.4 for the other set (see also Doll et al., 2016). This enabled us to dissociate model-free control, i.e., the simple repetition of rewarded choice regardless of the transition, from model-based control, which also takes into account whether the second-stage reward was linked to a rare transition (model-based control would demand a switch to the other option at the first-stage after a rare transition). Additionally, women were genotyped for the COMT-Val158Met polymorphism, a proxy of prefrontal dopamine content, in order to further examine the potentially non-linear relationship between estradiol and model-based control. Since none of the many previous studies on model-based learning controlled for the hormonal state of female subjects nor did they assess the interaction of estradiol with baseline dopamine content, the present study is the first to provide evidence regarding the role of endogenous estradiol in higher-order reinforcement learning.

## Materials and Methods

### Sample

In this study, 41 healthy young women [mean age (±SEM) = 24.6 ± 0.5 years; age range = 20–30 years], were tested with a TS-task in the early follicular phase, when circulating estradiol levels were low, and the late follicular phase, when circulating estradiol levels were high. Women were free of medication and hormonal contraceptives. For the 26 women who had previously taken hormonal contraceptives the mean distance of the first test day to the last intake of hormonal contraception was 15.8 months (SEM = 2.5 months; range = 2–36 months). Four women had stopped the intake 2 months before participation.

Women were included in the study if they had regular menstrual cycles and no gynecological problems, like polycystic ovary syndrome or endometriosis, or any other chronic disorder of the hormone system, e.g., Diabetes, Hashimoto’s thyroiditis. Current or previous psychiatric or neurological problems precluded study enrollment as did the present use of hormonal contraceptives. Subjects were of Middle European origin as determined by the place of birth of their parents and grandparents. All subjects gave written informed consent and were paid for participation. The present study was approved by the local Ethics Committee (*Ethikkommission der Ärztekammer Hamburg*).

The women were tested twice within the follicular phase of the menstrual cycle. One test occurred during the first 3 days following the onset of menstruation, i.e., the early follicular phase, which is characterized by low estradiol. The other one took place 2–3 days before expected ovulation in the late follicular phase, when estradiol approached its cyclic maximum. For determination of the actual test day participants stated their average cycle length based on previous menstrual cycles. Upon the onset of menstrual bleeding (cycle day 1) we then used the average cycle length to calculate the last expected cycle day (anticipated cycle end) in the given menstrual cycle. This enabled us to determine the optimal test day with a common counting method: For all subjects with an average cycle length shorter than 28 days, we subtracted 15 days from the anticipated cycle end. For subjects with an average of 28–31 days, 16 days were subtracted, and for cycle lengths longer than 31 days, 17 days were subtracted to schedule the late follicular phase test. Our subjects also tracked the daily concentration of the gonadotrophin Lutropin, which experiences a steep rise approximately 36 h prior to ovulation. For this, a common urine test (One Step^®^ by AIDE Diagnostic Co., Ltd.) was used. The urine test was performed on a daily basis starting 2 days before the scheduled late follicular phase test. In case of a positive result either before or on the day of the scheduled test, the behavioral test was postponed to the subsequent menstrual cycle. Test order was balanced between subjects and half of the subjects started in the early follicular phase. We initially recruited 48 women for the study. Of these, seven women dropped-out after completion of the first test day. Therefore the test order of the repeated tests was slightly biased toward the early follicular phase (24 women started in the early follicular phase). There was no significant interaction between test order and cycle phase when assessing the two learning components as shown in Table 1.

**TABLE 1:** Results of the repeated-measures ANOVAs with the factors “cycle phase” and “test-order” separately for drift and stable phase.

Main effect or interaction	*F*-value	df	*p*-value	Partial eta^2^
*(A) Drift-phase – model-free component*				
Cycle phase*	**7.45**	**1, 39**	**0.009**	**0.16**
Test order	0.002	1, 39	0.969	<0.01
Cycle phase × test order	2.60	1, 39	0.115	0.06
*(B) Stable-phase – model-free component*				
Cycle phase	0.13	1, 39	0.719	<0.01
Test order*^,1^	**4.24**	**1, 39**	**0.046**	**0.10**
Cycle phase × test order	0.84	1, 39	0.365	0.02
*(C) Drift-phase – model-based component*				
Cycle phase	0.05	1, 39	0.828	<0.01
Test order	0.88	1, 39	0.355	0.02
Cycle phase × test order	0.29	1, 39	0.594	0.01
(*D) Stable-phase – model-based component*				
Cycle phase	0.21	1, 39	0.648	<0.01
Test order	2.66	1, 39	0.111	0.06
Cycle phase × test order	0.32	1, 39	0.573	<0.01

*Significant effects (p < 0.05) are plotted in bold and are marked with an asterisk. ^1^The direct comparison of subjects who started their first test in the early follicular phase (early-to-late group; n = 24) with those that started in the late follicular phase (late-to-early group; n = 17) yielded a significant difference in the model-free score of the stable phase (mean ± SEM: early-to-late = 20.6 ± 2.7; late-to-early = 11.8 ± 3.4; t_(39)_ = 2.06, p = 0.046). This suggests that one group of participants used the model-free learning component to a greater extent, yet this effect was independent of cycle phase. This was also supported by the exploratory analysis of the respective test days. It showed that subjects from the early-to-late group, who were in the late follicular phase on the second test day, had a trend-wise higher model-free score than subjects in the early follicular phase (2nd test day: Early = 9.8 ± 4.1; Late = 19.8 ± 3.5; t_(39)_ = −1.86, p = 0.071). Similarly, on the first test day the subjects from the early-to-late group being in the early follicular phase now had a somewhat higher score on that day, even though again this difference was not significant (1st test day: Early = 21.5 ± 3.0; Late = 13.9 ± 4.6; t_(39)_ = 1.46, p = 0.153).

### Collection and Analysis of Salivary Estradiol

On each test day, subjects collected five samples of morning saliva at home. Starting at their normal wake-up time, each subject collected the samples (2 ml Eppendorf tubes) at regular intervals over 2 h, in order to control for the episodic secretion pattern of steroid hormones. During the sampling period, no consumption of food or beverages other than water was allowed to avoid sample contamination. Also, 12 h before sample collection subjects refrained from eating meat or other animal products. On the same day, the participants brought the samples to the lab, where they were immediately frozen at −20°C until further analyses. The subsequent analysis of free estradiol content was based on the aliquots of the five samples and used a 17beta-Estradiol Luminescence Immunoassay (IBL International, Tecan Group, Hamburg, Germany). The analysis followed the instructions provided by the manufacturer. Altogether, this allowed us to analyze the salivary estradiol level from the repeated tests of 39 subjects. The remaining samples of two women could not be analyzed as the two Immunoassay-plates we used each provided only 39 wells for double sampling.

### DNA Collection, Extraction, and Genotypic Analysis

Genotyping was performed by a commercial laboratory (Bioglobe, Hamburg, Germany). DNA was extracted from buccal swabs and purified with a standard commercial extraction kit. The analysis of the single nucleotide polymorphisms (SNP) rs4680 was performed on the MassARRAY^®^ system (Agena Bioscience) applying the iPLEX^®^ method and MALDI-TOF mass spectrometry for analyte detection. In general, all iPLEX reactions were performed according to the standard protocol recommended by the system supplier. The protocol generates allele-specific analytes in a primer extension reaction applying a primer directly adjacent to the SNP site. Assay design was performed with platform-specific software for the SNP sequences, aided by database information accounting for homologous regions and annotated secondary sequence variations in close proximity to the target SNP (proxSNPs). Based on rs-IDs, the multiplex assay design was performed with MassARRAY assay design suite v2.0. The final *in silico* design output was composed of a single multiplex reaction (8plex). The PCR amplification procedure used the following two primers: ACGTTGGATGTTTTCCAGGTCTGACAACGG and ACGTTGGATGACCCAGCGGATGGTGGATTT. The iPLEX primer was tCATGCACACCTTGTCCTTCA. The distribution of genotypes was in Hardy-Weinberg equilibrium (*p* = 0.18; two-tailed) as determined by the HW-Quick Check software by Steven T. Kalinowski^1^.

Altogether, four participants were homozygote for the Val allele (Val/Val), while 25 subjects were heterozygote (Met/Val), and 12 subjects were homozygote for the Met allele (Met/Met). Based on the distribution of genotypes, we decided to combine the Val/Val and Met/Val, who constitute the group of “Val-carriers” in all subsequent analyses. The two groups, Met/Met and Val-carriers did not differ in most demographic characteristics like average cycle length, cycle day of early and late follicular test, estradiol level on the respective test day, and trait impulsiveness as determined by Barratt Impulsiveness Scale (BIS-11) (Patton et al., 1995), however, Met allele homozygotes were slightly older than Val allele carriers (see Table 2).

**TABLE 2:** Demographic data divided by genotype.

	Met allele homozygotes	Val allele carriers	
	Mean ± SEM	Mean ± SEM	*t*-value (*p*-value)
Age (years)*	26.6 ± 0.7	23.8 ± 0.5	**2.95 (0.005)**
Mean length of two consecutive menstrual cycles (days)	29.2 ± 1.1	30.7 ± 0.7	−1.18 (0.245)
Cycle day of early follicular phase	1.8 ± 0.4	2.5 ± 0.3	−1.29 (0.206)
Cycle day of late follicular phase	13.4 ± 0.8	13.2 ± 0.4	0.20 (0.839)
Estradiol level of early follicular phase (pg/ml)	2.98 ± 0.63	3.00 ± 0.27	−0.04 (0.969)
Estradiol level of late follicular phase (pg/ml)	3.81 ± 0.60	4.56 ± 0.35	−1.11 (0.274)
Impulsiveness score (BIS-11)	60.9 ± 2.5	62.7 ± 1.4	−0.64 (0.526)

*Significant differences (p < 0.05) are plotted in bold and are marked with an asterisk.

### Task Description

Participants started the Two-Step task with a computer-based tutorial and a short training of 20 trials, which was supervised by the experimenter. Then the sequential TS-task with 300 trials in total was performed. Participants were tested with the version of the TS-task already employed by Doll et al. (2016). The TS-task incorporates a two-stage choice structure to achieve positive feedback (a virtual 1 Euro-coin) and tests for the individual model-free and model-based learning capacity. It thus captures the distinction between model-free learning behavior, i.e., the ability to adapt behavior based on direct performance feedback, and prospective model-based learning (Daw et al., 2005; Dolan and Dayan, 2013). In the first step of the TS-task, participants choose between two options (two Sanskrit symbols) within a time window of 2 s, which stochastically determines another set of choices with fixed transition probabilities between steps (i.e., 0.7 and 0.3, see Doll et al., 2016).

In the TS-task, model-free control of behavior describes the aspect of learning that increments the value of choices based on the outcome that directly follows, and regardless of the transitions experienced between task stages. In contrast, model-based control takes the history of outcomes as well as the noisy task structure prospectively into account (Daw et al., 2005). Model-based learning is particularly important in the TS-task, since an actual reward can only be reached after the two consecutive choices. The first stage choice thereby determines with a certain probability, which pair of options is available for the second stage choice. For each action at the first stage, one pair of options at stage 2 is more likely to occur (common transition), while the other pair is less likely (rare transition). The model-based component is assumed to take these transition probabilities into account, while the model-free component is not. From this, certain predictions can be made: In case of a rare transition, the model-free component would use the feedback at stage 2 to choose the stage 1 stimulus independent of the nature of the transition. It would stay with the previous first-stage choice, even after a rare second-stage reward, which would lower overall reward outcome. In contrast, after receiving a rare reward, model-based control would probably bias the decision toward a switch at stage 1 and the choice of the option more likely to transition to the second-stage state that would have produced reward on the last trial (switch to the common transition) (Doll et al., 2016). Based on these predictions and the stay frequencies from stage 1, we calculated the model-based and model-free learning components according to Sebold et al. (2014), which could then be compared between cycle phases.

The model-free score thereby reflected the main effect of reward on stay frequencies that was calculated by:

$$\begin{array}{c} model-freescore=\%rewardedcommontransition\\ +\%rewardedraretransition-\%unrewardedcommon\\ transition-\%unrewardedraretransition \end{array}$$

The model-based score mirrored the interaction between transition frequency and reward, which was indicated by:

$$\begin{array}{c} model-basedscore=\%rewardedcommontransition\\ +\%unrewardedraretransition-\%rewardedraretransition\\ -\%unrewardedcommontransition \end{array}$$

In contrast to other versions of the TS-task, the specific version employed by Doll et al. (2016) included two task phases, the drift phase of the first 150 trials and the stable phase of the remaining 150 trials, which were characterized by different degrees of reward uncertainty at the second stage choice. During the drift phase the second stage choice is followed by reward with a slowly and randomly drifting probability set within the boundaries of 0.25 and 0.75. In the present study, one of four sets of drifts was randomly assigned to each person in each cycle phase, whereby the assignment did not differ between cycle phases or COMT genotypes (*p* > 0.39). The design feature of the drift phase emphasized model-free updating, as subjects learned the values of these stimuli incrementally. In the remaining 150 trials (the stable phase) the reward probabilities reached their final values of 0.7 versus 0.3 in state 1, and 0.6 versus 0.4 in state 2.

### Statistical Analysis

First, we analyzed the individual stay frequencies at the first stage choice with a repeated-measures analysis of variance (ANOVA). This was done separately for the drift and the stable phase in order to account for the different degrees of reward uncertainty (see task description above). The ANOVA assessed stay frequencies in relation to the reward achieved at stage 2 of the previous trial, i.e., the factor “*previous reward*” (yes, no), and the previous transition that led to this reward, i.e., factor “*previous transition*” (rare or common), as well as their interaction. Additionally, the ANOVA also included the within-subject factor “*cycle phase*” (early or late follicular phase) and the between-subjects factor “*COMT genotype*” (Val-carriers, Met-homozygotes). The effect size is reported as partial eta^2^. *Post hoc* tests used paired or independent *t*-tests. For effect sizes we use Cohen’s *d* or Hedge’s *g* for comparisons including one group with *n* < 20. Statistical significance was assumed at *p* < 0.05, two-tailed, if not indicated otherwise.

In a second step, we looked more specifically at differences in the model-free and model-based learning components. For this, we calculated the model-based and model-free learning components according to Sebold et al. (2014) (see above), which were then compared between cycle phases and genotypes, respectively.

## Results

### Analysis of Menstrual Cycle Phase Related Changes in Estradiol Level

Estradiol level followed the predicted cycle-typical pattern and significantly increased from the early to the late follicular phase [mean ± SEM: estradiol_early_ = 2.99 ± 0.26 pg/ml; estradiol_late_ = 4.35 ± 0.31 pg/ml; *t*_(38)_ = 4.48, *p* < 0.001, one-tailed], also within the subgroup of Val-carriers [*t*_(27)_ = 4.03, *p* < 0.001, one-tailed] and in the Met-homozygotes [*t*_(10)_ = 2.06, *p* < 0.034, one-tailed]. In that way, the early follicular and the late follicular phase can be considered as the low and the high estradiol state, respectively.

### Analysis of Stay Frequencies and Learning Scores of the Drift Phase

In the drift phase, the choice at stage 2 was followed by probabilistic reward with a slowly and randomly drifting probability. Thus, the drift phase required a constant updating of the current decision to maximize reward, like in other versions of the TS-task previously employed (e.g., Deserno et al., 2015; Doll et al., 2016; Kroemer et al., 2019).

**FIGURE 1:**
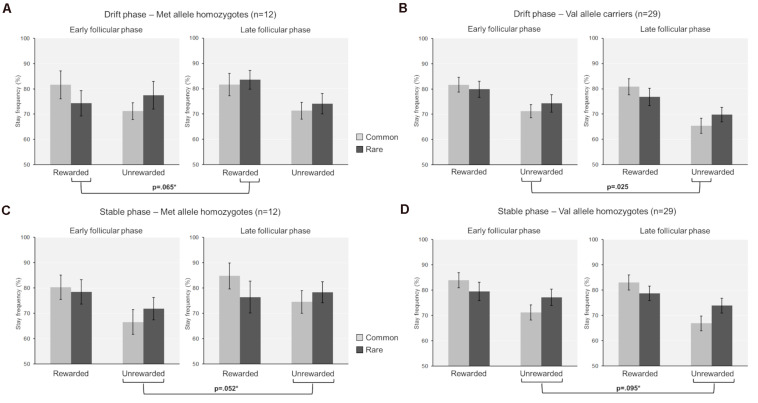
Mean stay frequencies separated by task phase (drift versus stable phase), genotype group (Met-homozygotes versus Val-carriers) and cycle phase (early versus late follicular phase). **(A)** Drift phase, Met-homozygotes. **(B)** Drift phase, Val-carriers. **(C)** Stable phase, Met-homozygotes. **(D)** Stable phase, Val-carriers. The differences between cycle phases are indicated with the respective *p*-value. These also include statistical trends (*p* < 0.10), for which the actual delta-values of stay frequencies (Delta stay frequency_late vs. early follicular phase_) were significantly different between the genotypes (delta values are not shown here, but are reported in the text). These statistical trends are additionally marked with an asterisk, if the direct comparison between the genotypes yielded a significant difference (*p* < 0.05).

First, we assessed the influence of hormonal state and genetic variance on the stay frequencies at the first stage choice of the drift phase. The stay frequencies thereby represent the probability that the same stage 1 choice would be made on the next trial. We identified a significant main effect of “previous reward” [*F*_(1,39)_ = 39.63, *p* < 0.001, partial eta^2^ = 0.5] and a significant interaction of “previous reward” by “previous transition” [*F*_(1,39)_ = 9.80, *p* = 0.003, partial eta^2^ = 0.2], indicating that participants used both model-free and model-based learning while performing the task (Daw et al., 2011). We also found a significant two-way interaction between “cycle-phase” and “previous reward” [*F*_(1,39)_ = 6.56, *p* = 0.014, partial eta^2^ = 0.14]. This was reflected by enhanced avoidance of non-reward *per se* in the late as opposed to the early follicular phase [non-reward: stay frequency_early_ ± SEM = 73.2 ± 2.3%; stay frequency_late_ ± SEM = 69.1 ± 2.1%; *t*_(40)_ = 2.23, *p* = 0.031, *d* = 0.33]. In addition to that, a four-way interaction between “cycle-phase,” “previous reward,” “previous transition,” and “COMT genotype” was found [*F*_(1,39)_ = 4.48, *p* = 0.041, partial eta^2^ = 0.1] (see also Table 3 for the complete ANOVA results). Accordingly, the Val-carriers became better at avoiding the commonly non-rewarded option in the late follicular phase (stay frequency_late_ ± SEM = 65.4 ± 3.0%) compared to the early follicular phase [stay frequency_early_ ± SEM = 71.2 ± 2.6%; *t*_(28)_ = 2.18, *p* = 0.038, *d* = 0.44]. Since the Val-carriers represented the majority of the test group, this change probably drove the above described two-way interaction between “cycle-phase” and “previous reward.” Apart from that, we also observed that the stay frequencies of the Met-homozygotes in relation to rare reward showed a trend-wise increase in the late follicular phase [stay frequency_early_ ± SEM = 74.3 ± 5.0%; stay frequency_late_ ± SEM = 83.5 ± 3.8%; *t*_(11)_ = −2.04, *p* = 0.065, *d* = 0.53]. This increase was also significantly different from the delta observed in Val-carriers [Delta stay frequency_late vs. early_ ± SEM: Met/Met = 9.2 ± 4.5%; Val carriers = −3.1 ± 2.8%; *t*_(39)_ = 2.35, *p* = 0.025, Hedge’s *g* = −0.81], suggesting that Met-homozygotes became impaired in their ability to adequately integrate the complex task structure in their choices when being in the high estradiol state (see Figures 1A,B).

**TABLE 3:** Drift phase – Effects of cycle-phase, TS-task manipulation and COMT-genotype on stay frequencies.

Main effect or interaction	*F*-value	df	*p*-value	partial eta^2^
Previous reward*	**39.63**	**1, 39**	**<0.001**	**0.50**
Previous transition	0.58	1, 39	0.450	0.02
Cycle phase	0.37	1, 39	0.546	0.01
COMT genotype	0.17	1, 39	0.680	<0.01
Previous reward × previous transition*	**9.80**	**1, 39**	**0.003**	**0.20**
Previous reward × cycle phase*	**6.56**	**1, 39**	**0.014**	**0.14**
Previous reward × COMT genotype	1.25	1, 39	0.270	0.03
Previous transition × cycle phase	0.29	1, 39	0.596	0.01
Previous transition × COMT genotype	0.08	1, 39	0.780	<0.01
Cycle phase × COMT-genotype	2.238	1, 39	0.143	0.05
Previous reward × previous transition × cycle phase	1.39	1, 39	0.245	0.03
Previous reward × previous transition × COMT genotype	0.02	1, 39	0.899	<0.01
Previous reward × cycle phase × COMT genotype	0.70	1, 39	0.408	0.02
Previous transition × cycle phase × COMT genotype	0.53	1, 39	0.469	0.01
Previous reward × previous transition × cycle phase × COMT genotype*	**4.48**	**1, 39**	**0.041**	**0.10**

*Significant effects (p < 0.05) are plotted in bold and are marked with an asterisk.

**FIGURE 2:**
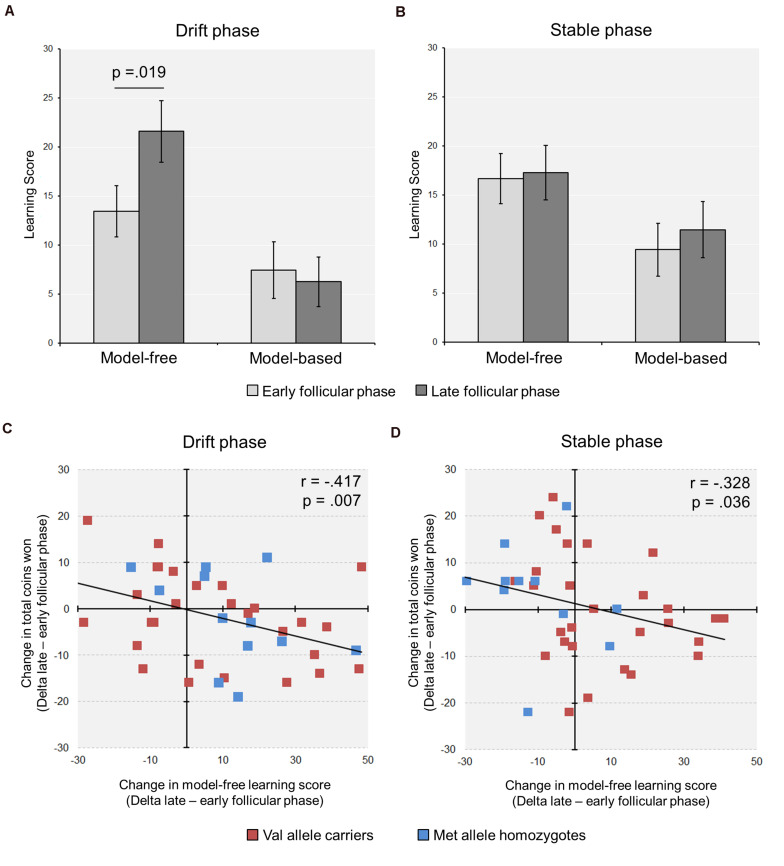
Cycle-phase modulates model-free learning in the Two-Step task (*n* = 41). **(A)** During the drift phase, a significant increase in model-free learning from the early to the late follicular was observed, while no change in model-based learning occurred. **(B)** During the stable phase, with fixed reward probabilities at stage two, the learning scores remained unchanged between cycle phases. **(C,D)** The relative increase in model-free learning from the early to the late FP was associated with a reduction in the relative amount of coins won, i.e., Δpoints (late minus early follicular phase), in both the drift **(C)** and the stable phase **(D)** (For display purposes, the individual data points of the Met/Met homozygotes and the Val allele carriers are shown in different colors).

In a second step, we calculated the model-free and the model-based scores based on the stay frequencies (Sebold et al., 2014). We found a significant increase in the model-free score from the early to the late follicular phase in the complete group of subjects [Drift phase: model-free_early_ ± SEM = 15.51 ± 2.18; model-free_late_ ± SEM = 19.81 ± 2.65; *t*_(40)_ = −2.44, *p* = 0.019, *d* = −0.44] (see Figure 2A). Notably, the relative increase in model-free learning from the early to the late follicular phase (Delta_model–free_) was related to more suboptimal decision making, reflected by a reduced task success in terms of the total number of acquired coins during the drift phase (*r* = −0.417, *p* = 0.007, *n* = 41) (see Figure 2C).

**FIGURE 3:**
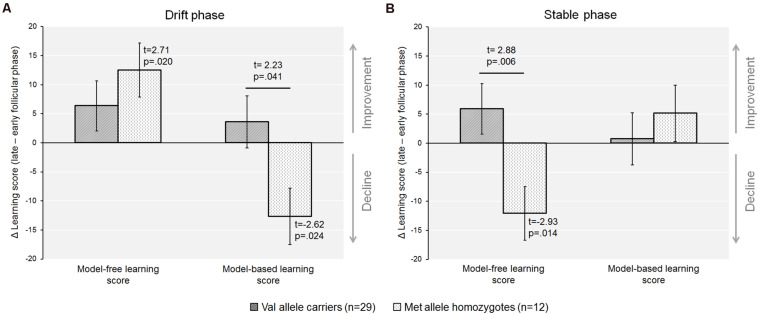
The recruitment of model-free and the model-based control varied between cycle phases when accounting for COMT-Val158Met genotype. **(A)** In the drift phase, Met-homozygotes exhibited a significant decline in model-based control from the early to the late follicular phase, whereas Val-carriers remained unaffected by cycle phase. Further, the Δ_(late minus early follicular phase)_ for both the model-based and the model-free score differed from zero in the Met/Met genotype only, indicating that individuals with higher prefrontal dopamine were apparently more negatively affected by the rise in endogenous estradiol. **(B)** In the stable phase, there was a relative decline in model-free control in Met-homozygotes only, that was also significantly different in the comparison of genotypes.

When the sample was dichotomized by genotype we found that the increase in the model-free score from the early to the late follicular phase was only significant in the Met-homozygotes [model-free_*early*_ ± SEM = 7.2 ± 5.4; model-free_late_ ± SEM = 19.7 ± 5.8; *t*_(11)_ = −2.71, *p* = 0.020, *d* = −0.65], but not in Val-carriers [model-free_*early*_ ± SEM = 16.1 ± 2.9; model-free_late_ ± SEM = 22.4 ± 3.8; *t*_(28)_ = −1.47, *p* = 0.15]. In addition, Met-homozygotes also showed a concurrent decline of the model-based score during the drift phase [model-based_early_ ± SEM = 13.6 ± 6.0; model-based_late_ ± SEM = 0.9 ± 3.2; *t*_(11)_ = 2.62, *p* = 0.024, *d* = 0.67], which was again absent in Val-carriers [model-based_early_ ± SEM = 4.9 ± 3.2; model-based_late_ ± SEM = 8.5 ± 3.2; *t*_(28)_ = −0.80, *p* = 0.431]. The magnitude of cycle phase related changes in both the model-free and model-based learning scores, i.e., the Delta value of the score from the late minus the early follicular phase, was also significantly different from zero in the Met-homozygotes (see Figure 3A; see also Table 4).

**TABLE 4:** Comparison of model-free and model-based scores between genotype and cycle phases.

	Early follicular phase (mean ± SEM)		Late follicular phase (mean ± SEM)		Independent *t*-test	Paired *t*-test
				
	Met/Met (*n* = 12)	Val-carriers (*n* = 29)	Met/Met (*n* = 12)	Val-carriers (*n* = 29)	Between genotypes, within cycle phases	Between cycle phases, within genotype
**Drift phase**						
Model-free score	7.2 ± 5.4	16.1 ± 2.9	19.7 ± 5.8	22.4 ± 3.8	n.s.	**Met/Met**: *t* = −2.71 *p* = 0.020
Model based score	13.6 ± 6.0	4.9 ± 3.2	0.9 ± 3.2	8.5 ± 3.2	n.s.	**Met/Met**: *t* = 2.62 *p* = 0.024
**Stable phase**						
Model-free score	20.4 ± 4.6	15.1 ± 3.1	8.4 ± 5.6	21.0 ± 3.0	**Late:** *t* = −2.16 *p* = 0.037	**Met/Met**: *t* = 2.93 *p* = 0.014
Model based score	7.1 ± 5.0	10.4 ± 3.3	12.2 ± 6.9	11.2 ± 2.9	n.s.	n.s.

### Analysis of Stay Frequencies and Learning Scores of the Stable Phase

Following the drift phase, unbeknownst to participants, the reward probabilities at stage two stopped drifting and remained fixed. In principle, this stable phase requires a lower learning rate and the difficulty of learning is reduced, since reward probabilities are reliable now. During the stable part of the TS-task, we also found a significant main effect of “previous reward” [*F*_(1,39)_ = 45.22, *p* < 0.001, partial eta^2^ = 0.54] and a significant interaction of “previous reward” by “previous transition” [*F*_(1,39)_ = 18.60, *p* < 0.001, partial eta^2^ = 0.32]. However, in contrast to the drift phase, we observed a significant three-way interaction between “cycle phase”, “previous reward” and “COMT genotype” [*F*_(1,39)_ = 8.30, *p* = 0.006, partial eta^2^ = 0.18] (see also Table 5 for a complete list of the ANOVA results). This was reflected by a differential change in avoidance learning capacity between cycle phases and genotypes. We found a reduced avoidance capacity of non-reward (i.e., a higher stay frequency for non-reward) in the late relative to the early follicular phase in Met-homozygotes compared to the Val-carriers [Delta stay frequencies_late–early_ ± SEM: Met/Met = 7.27 ± 3.35%; Val-carriers = −3.8 ± 2.19%; *t*_(39)_ = 2.74, *p* = 0.009, Hedge’s *g* = 0.94]. Further conforming to this pattern, the direct comparison of cycle phases within genotype groups revealed two statistical trends, with the Met-homozygotes showing a slight reduction in avoidance learning capacity [stay frequency_non–reward_ ± SEM: Early = 69.2 ± 4.2%; Late = 76.5 ± 3.8%; *t*_(11)_ = −2.17, *p* = 0.052, *d* = 0.60], while the Val-carriers showed a trend-wise increase in this capability [non-reward stay frequency ± SEM: Early = 74.2 ± 3.0%; Late = 70.4 ± 2.7%; *t*_(28)_ = 1.73, *p* = 0.095, *d* = 0.31] (see Figures 1C,D).

**TABLE 5:** Stable phase – Effects of cycle-phase, TS-task manipulation and COMT-genotype on stay frequencies.

Main effect or interaction	*F*-value	df	*p*-value	partial eta^2^
Previous reward*	**45.22**	**1, 39**	**<0.001**	**0.54**
Previous transition	0.19	1, 39	0.663	0.01
Cycle phase	0.26	1, 39	0.611	0.01
COMT genotype	0.01	1, 39	0.939	<0.01
Previous reward × previous transition*	**18.60**	**1, 39**	**<0.001**	**0.32**
Previous reward × cycle phase	0.97	1, 39	0.330	0.02
Previous reward × COMT genotype	0.58	1, 39	0.452	0.02
Previous transition × cycle phase	0.52	1, 39	0.474	0.01
Previous transition × COMT genotype	0.56	1, 39	0.460	0.01
Cycle phase × COMT-genotype	3.01	1, 39	0.091	0.07
Previous reward × previous transition × cycle phase	0.55	1, 39	0.461	0.01
Previous reward × previous transition × COMT genotype	0.06	1, 39	0.812	<0.01
Previous reward × cycle phase × COMT genotype*	**8.30**	**1, 39**	**0.006**	**0.18**
Previous transition × cycle phase × COMT genotype	1.04	1, 39	0.315	0.03
Previous reward × previous transition × cycle phase × COMT genotype	0.31	1, 39	0.583	0.01

*Significant effects (p < 0.05) are plotted in bold and are marked with an asterisk.

With regard to the learning scores, the stable phase yielded partly different results than the drift phase. First, in the analysis of the complete group the model-free score remained unaffected by cycle phase [model-free score ± SEM: Early = 16.7 ± 2.6; Late = 17.3 ± 2.8; *t*_(40)_ = −0.21, *p* = 0.835], like the model-based score [model-based score ± SEM: Early = 9.4 ± 2.7; Late = 11.5 ± 2.9; *t*_(40)_ = −0.57, *p* = 0.572] (see Figure 2B). However, similar to the drift phase the increased model-free control in the late follicular phase negatively correlated with the delta of totally acquired coins in the 150 trials of the stable phase (*r* = −0.328, *p* = 0.036, *n* = 41) (see Figure 2D).

Secondly, when separately looking at the two genotypes we found that model-free processing decreased in Met-homozygotes in the high estradiol state [model-free_early_ ± SEM = 20.4 ± 4.6; model-free_late_ ± SEM = 8.4 ± 5.6; *t*_(11)_ = 2.93, *p* = 0.014, *d* = 0.67]. Additionally, this strong decline in model-free processing capacity in the Met-homozygotes (Delta_late–early_ = −12.1 ± 4.1) differed from the delta of the Val-carriers [Delta_late–early_ = 5.9 ± 3.6; *t*_(39)_ = −2.88, *p* = 0.006, Hedge’s *g* = 0.99]. In contrast to that, the model-based component remained unchanged in the Met-homozygotes [model-based score ± SEM: Early = 7.1 ± 5.0; Late = 12.2 ± 7.0; *t*_(11)_ = −0.76, *p* = 0.462].

Finally, similar to the drift phase, the Val-carriers did not show significant cycle-related changes in the learning scores during the stable phase [model-free score ± SEM: Early = 15.1 ± 3.1; Late = 21.0 ± 3.0; *t*_(28)_ = −1.63, *p* = 0.114] [model-based score ± SEM: Early = 10.4 ± 3.3; Late = 11.2 ± 2.9; *t*_(28)_ = 0.176, *p* = 0.861] (see Figure 3B).

## Discussion

Variations in estradiol may influence dopaminergic transmission and basic (model-free) aspects of reinforcement learning as well as higher-order cognition (Jacobs and D’Esposito, 2011; Becker, 2016; Diekhof and Ratnayake, 2016). Here, we examined whether changes in estradiol modulate both model-free and model-based reinforcement learning across the menstrual cycle, also depending on the COMT-Val158Met genotype. The results showed that women relied more heavily on model-free learning in the high compared to the low estradiol state, yet only when reward associations were volatile. This suggests that the increased estradiol level may have led to a disruption of frontostriatal interactions during reinforcement learning. This seems plausible, since estradiol inhibits both striatal DRD2 expression and prefrontal COMT activity, which should interfere with the prospective updating of value representations in the striatum and should reduce the prefrontal signal-to-noise ratio during the maintenance of behavioral goals. At the same time, estradiol enhances dorsolateral striatal dopamine transmission through DRD1, which would also favor habitual model-free control (Lévesque et al., 1989; Xie et al., 1999; Schendzielorz et al., 2011; Krentzel and Meitzen, 2018; see also Becker, 2016; Yoest et al., 2018 for review). When further accounting for individual differences in the prefrontal dopaminergic baseline capacity, we observed that Met-homozygotes with high prefrontal dopamine also experienced a decline in model-based control in the context of volatile reward probabilities. In contrast, the model-based score of Val-carriers remained unaffected by menstrual cycle phase. Altogether, these initial findings lead us to infer that the endogenous change in estradiol does not only affect model-free control, but also modulates prospective model-based learning depending on prefrontal baseline capacity.

We observed an increase in the propensity to use model-free control in the high estradiol state in the complete group of our subjects, yet only when reward-outcome was volatile. The increase in model-free control was thereby related to reduced task performance (reduced task success in terms of the total number of acquired coins), suggesting that the predominant use of model-free control was suboptimal for reward maximization in the TS-task. It has been suggested that model-free learning may be primarily mediated by striatal processing, whereas model-based control may recruit both striatal and prefrontal resources (Deserno et al., 2015; Doll et al., 2016). Interestingly, the effect appeared to be specifically driven by the Met-homozygotes, who showed an increase in model-free control as well as a concurrent reduction in the capacity for model-based learning during the drift phase, while the Val-carriers showed no significant change in learning capacity. In that way, the present observations may conform with the notion that higher estradiol could have biased striatal processing toward the model-free, less flexible learning component, and might even have concurrently disrupted frontostriatal interactions necessary for model-based control, at least in the Met-homozygotes. In the striatum of female rodents, estradiol increases stimulated dopamine release, particularly so in the dorsolateral striatum (Becker, 2016). In our study, estradiol may thus have disrupted the balance between model-based and model-free control by favoring model-free processing and the incentive salience of immediate reward during the drift phase. This becomes particularly likely when also considering the environmental volatility of the drift phase. In their theoretical paper on partial reinforcement, Anselme (2015) proposed that incentive motivation may outweigh the effect of actual learning on behavioral choice when a reward outcome is uncertain. In humans, reward uncertainty increases tonic dopamine in the midbrain and promotes reward-related ventral striatal activation (Dreher et al., 2006). One may therefore assume that the combined effect of reward volatility and high estradiol could have biased behavioral choice toward model-free control. In fact, Met-homozygotes also showed an increased stay frequency following rare reward, which could have reflected such a maladaptive increase in the incentive salience of immediate reward.

Our observation of the estradiol-driven increase in model-free control during the drift phase does neither fit with the previously reported result of a disruption of model-free control by the dopamine agonist L-DOPA (Kroemer et al., 2019), nor with another observation of no influence of L-DOPA on model-free learning, yet a positive effect on model-based control (Wunderlich et al., 2012). However, these studies differ in some important aspects from our own: First, any differences to our young female sample (*n* = 41 women) could have been related to the male predominance in the other two samples [Wunderlich et al. (2012) tested 18 young male undergraduates (mean age = 23 years), and Kroemer et al. (2019) examined a representative adult sample (mean age = 37 years) of 49 men and 16 women], and might therefore reflect biological sex differences in the mechanisms underlying reinforcement learning (see Becker, 2016; Diekhof, 2018). Second, estradiol and L-DOPA modulate different dopaminergic mechanisms. Whereas, L-DOPA increases dopaminergic tone (Harun et al., 2016) and thus reduces local dopamine changes after unexpected reward, estradiol facilitates stimulated dopamine release (Becker, 1990, 1999; Xiao and Becker, 1998; Hu et al., 2006). More specifically, in the prefrontal cortex, estradiol reduces tonic dopamine, yet augments transient dopamine release following stimulation, whereas in the striatum it increases both tonic and phasic dopamine (Almey et al., 2015). Therefore, estradiol would probably increase dopaminergic transmission after unexpected reward, which would in turn increase model-free control, as presently observed.

Only Met-homozygotes exhibited a compromised model-based learning capacity during the drift phase when being in the late follicular phase. This was expressed by an increase difficulty in the differentiation between common and rare reward, with higher maladaptive stay frequencies after rare rewards. These observations fit with the assumption that, on the one hand, being homozygote for the Met allele is beneficial for the stabilization of prefrontal information processing and may protect goal-directed information from interference, since it may keep the optimal range of dopamine for cognitive processing (Durstewitz and Seamans, 2008; Slifstein et al., 2008; Schacht, 2016). On the other, the estradiol-promoted increase of prefrontal dopamine should then have destabilized information processing, also by disrupting the overall frontostriatal balance (Durstewitz and Seamans, 2008). Even though our sample included only 12 Met-homozygotes, the observed decline in model-based learning during the state of increased reward uncertainty may in fact correspond to this pattern. Jacobs and D’Esposito (2011) found a similar interaction between estradiol and COMT genotype in a working memory task. In their study the 8 Met-homozygotes showed a performance decline and a reduction of prefrontal activation while processing the cognitively demanding lure trials of an N-back task in the late follicular phase. Conversely, in their study the 13 women homozygote for the Val-allele apparently benefited from the higher estradiol and showed enhanced cognitive performance, while prefrontal activation was concurrently increased. In the present study, we did not find a state-related change in the model-based learning component of the 29 Val-carriers. We can only speculate that the predominance of heterozygotes in this group (only 4 Val-homozygotes) may explain this finding. Heterozygosity may place an individual somewhere near or even within the optimal range of prefrontal dopamine (Schacht, 2016) and it could be expected that perturbations of dopamine through an endogenous agonist such as estradiol may not at any case move an individual beyond this range.

Further notably, the decline in model-based learning in the Met-homozygotes was restricted to the state of increased environmental volatility. We assume that this might have been the result of the combined influences of (1) increased task familiarity, and (2) the concurrent reduction of task difficulty. Task familiarity, which can be achieved through extensive training, may automatize model-based learning in the TS-task. Economides et al. (2015) showed that repeated performance of the TS-task on two consecutive days preserved model-based control even in a dual-task condition. We assume that the reduced task difficulty and increased task familiarity rendered model-based learning less vulnerable to the influence of estradiol during the stable phase, even in Met-homozygotes. Further, previous evidence points toward a crucial involvement of striatal DRD2 in the updating of goal-relevant representations, especially in situations of increased task difficulty (Cools and D’Esposito, 2011). High estradiol can suppress DRD2-action and increases stimulated dopamine release (Krentzel and Meitzen, 2018; see also Becker, 1999; Yoest et al., 2018). Therefore, estradiol may particularly interfere with the ability to update changing value representations, which was crucially important for mastering the drift phase. If we further presume that cognitive load was increased by the volatile reward structure, we should also expect an additional load-dependent increase in dopamine (see also Mattay et al., 2003, who reported a similar interaction of increased cognitive load and the dopamine agonist amphetamine on working memory). This would also explain why the Met-homozygotes showed a decline in model-based control during the difficult drift, but not during the relatively easy stable phase.

In the stable part of the TS-task, we found that, in contrast to the drift phase, model-free control decreased from the early to late follicular phase in the Met/Met genotype, i.e., enhanced stay frequencies in relation to non-reward, yet regardless of transition type. Interestingly, this latter finding contrasted that of the Val-carriers, who in the high estradiol state became better at avoiding non-reward. Two previous studies found an interaction between estradiol and avoidance learning capacity. Diekhof and Ratnayake (2016) observed reduced activation of the dorsal anterior cingulate cortex to negative feedback and reduced avoidance learning performance in the late follicular phase. Jakob et al. (2018) reported a similar effect, yet only in subjects with a low striatal dopaminergic baseline. These observations fit with the stable phase result of the Met-homozygotes, but antagonize the observation in Val-carriers. Alternatively, the differences between genotypes may be explained by the interaction between dopamine and the prefrontal signal-to-noise ratio. Firstly, in humans the Val allele has been associated with a reduced prefrontal signal-to-noise ratio (Gallinat et al., 2003; Winterer et al., 2006a, b). Secondly, in rodents dopamine has been observed to increase the signal-to-noise ratio and promote the encoding of aversive stimuli in the medial prefrontal cortex (Weele et al., 2019). Thirdly, according to the inverted U-shape hypothesis the prefrontal deficit of Val-homozygosity can be transiently remedied, while the Met-homozygotes may be thrown out of balance by dopamine agonists (Cools and D’Esposito, 2011; Schacht, 2016). Since estradiol may down-regulate COMT activity (Xie et al., 1999; Schendzielorz et al., 2011), it should in turn increase prefrontal dopaminergic tone. Thus, in the dopamine-deficient Val-carriers higher estradiol might have increased the signal-to-noise ratio leading to a better avoidance of (common) non-reward in both phases (Cools and D’Esposito, 2011).

Nevertheless, this does not explain why Met-homozygotes showed such marked differences in model-free control between phases. We can only speculate that the marked differences in reward volatility might have involved dissimilar cognitive operations and thus taxed different physiological mechanisms to solve the task at hand. On the one hand, the drift phase was characterized by the need to learn stimulus values incrementally, making prospective learning less effective. This emphasized model-free learning from immediate outcome, particularly so in the high estradiol state, and because of the supposedly increased cognitive load, augmenting dopaminergic transmission (Becker, 1999, 2016; Mattay et al., 2003). On the other hand, stable reward contingencies and decreased task difficulty enabled the more effective use of model-based control in the second half of the TS-task. Although the ability to integrate non-reward into behavioral choice declined in the Met-homozygotes it did not impair overall gain in the stable phase. This indicates that the more effective use of model-based control outweighed the need to rely on model-free control of behavior here. In fact, the model-based system has been shown to cooperate with the model-free system and can “train” the latter by replaying and simulating experience offline. This may in turn allow for choice that appears model-based (see Gershman et al., 2014). Finally, it is possible that the behavioral adaptations to randomly drifting reward probabilities in combination with the increased effort the participants put into responding during the drift phase could have to some extent disguised an estradiol-related deficit in avoidance learning in the state of heightened estradiol.

## Conclusion

We found that cycle-related differences in reinforcement learning capacity were most pronounced during the state of increased environmental volatility (drift phase) and in Met-homozygotes, whose ability to use model-based learning was significantly reduced in the high estradiol state. Further, model-free learning appeared to be enhanced in the same state and this effect was already evident on the group level, but most pronounced in the Met/Met genotype. In contrast, Val-carriers remained widely unaffected by changes in endogenous estradiol. The present data suggest a disruption of frontostriatal interactions during reinforcement learning in a state of naturally enhanced estradiol. This seems plausible as estradiol may have an inhibitory influence on both striatal DRD2 expression and on prefrontal COMT activity, which should interfere with prospective updating of value representations in the striatum and reduce the prefrontal signal-to-noise ratio during the maintenance of behavioral goals. At the same time, estradiol may enhance dorsolateral striatal dopamine transmission through DRD1, which could decouple behavioral decisions from goal-directed, model-based choice and might favor model-free control. Consequently, the present observations may be important for the better understanding of mechanisms that lead to addiction and substance abuse or promote craving and relapse during abstinence in naturally cycling women.

## Data Availability Statement

The datasets presented in this study can be found in online repositories. The names of the repository/repositories and accession number(s) can be found in the article/Supplementary Material.

## Ethics Statement

The studies involving human participants were reviewed and approved by the Ethikkommission der Hamburger Ärztekammer. The patients/participants provided their written informed consent to participate in this study.

## Author Contributions

ED contributed to the conceptualization, methodology, investigation, validation, formal analysis, resources, data curation, visualization of results, project administration, project supervision, and wrote the original draft. MF was involved in the project supervision, methodology, and reviewed and edited the first draft. AG was involved in the methodology, formal analysis, and reviewed and edited the first draft. FO contributed to the formal analysis, investigation, data curation, and reviewed and edited the first draft. BD provided the task software and was involved in the methodology. All authors contributed to the article and approved the submitted version.

## Conflict of Interest

The authors declare that the research was conducted in the absence of any commercial or financial relationships that could be construed as a potential conflict of interest.

## Footnotes

1 http://www.montana.edu/kalinowski/software/hw-quickcheck.html
